# The Effect of 50% Dextrose on Serum Glucose Levels in Adult Hypoglycemic Patients: A Prospective Observational Study

**DOI:** 10.7759/cureus.80375

**Published:** 2025-03-10

**Authors:** Ahmed Al Saidi, Mohammed Al Shamsi, Abdulmunaim Al Farsi, Saif Al Tubi

**Affiliations:** 1 Emergency Medicine, Sohar Hospital, Sohar, OMN; 2 Emergency Medicine, Armed Forces Hospital, Muscat, OMN; 3 Emergency Medicine, The Royal Hospital, Muscat, OMN; 4 Emergency Medicine, Nizwa Hospital, Nizwa, OMN

**Keywords:** 50% dextrose, high-concentration glucose solution, hypoglycemia, rebound hyperglycemia, serum glucose level

## Abstract

Objective: This study aimed to observe changes in serum glucose levels following the administration of 50 mL of 50% dextrose solution (D50) in hypoglycemic patients. Specifically, we seek to determine the prevalence of rebound hyperglycemia episodes, the peak effect, and the duration of action of a single dose of 50 mL D50.

Method: This prospective observational study was conducted at four emergency departments (two tertiary care hospitals and two secondary care hospitals) in Oman over a three-year period, from May 1, 2019, to April 30, 2022. Adult patients (>12 years old) presenting with documented hypoglycemia (serum glucose level ≤3.9 mmol/L) were included. All patients underwent bedside serum glucose measurements at predetermined time intervals following the administration of D50. Data were analyzed using the Epidata program (Buenos Aires, Argentina). The mean serum glucose level at each time interval was calculated, along with the prevalence of rebound hyperglycemia. The independent sample t-test, one-way ANOVA, and Pearson's chi-squared test were used for statistical analysis.

Results: A total of 102 patients (53 females and 48 males) were included in this study. The majority of patients were diabetic (N = 87), while 15 (14.7%) were non-diabetic. Rebound hyperglycemia was predominantly observed within five minutes of administering 50 mL of D50, with a mean serum glucose level of 12.2 mmol/L and a maximum reading of 22.6 mmol/L. Rebound hyperglycemia occurred more frequently in the non-diabetic group (73.3%) compared to the diabetic group (56.3%). Six patients (5.7%) required a second dose of 50 mL D50, all of whom were diabetic.

Conclusion: A single dose of 50 mL D50 effectively restores and maintains the desired serum glucose level in non-diabetic hypoglycemic adult patients for up to 60 minutes. However, diabetic patients may require additional doses or continuous dextrose-containing fluids if they do not begin oral feeding. Using a lower concentration of dextrose-containing fluids may help mitigate the phenomenon of rebound hyperglycemia.

## Introduction

Hypoglycemia is a clinical syndrome characterized by low plasma glucose concentrations, resulting in various symptoms and signs that resolve once plasma glucose levels are normalized [1]. According to the American Diabetes Association (ADA) and the Endocrine Society, hypoglycemia is defined as any episode of abnormally low plasma glucose concentration (with or without symptoms) that exposes the individual to potential harm [2].

Symptomatic hypoglycemia is marked by low blood glucose levels accompanied by typical adrenergic symptoms such as sweating, palpitations, trembling, and tingling. Determining a single threshold value for plasma glucose concentration that correlates with the onset of hypoglycemia, particularly in patients with diabetes, is challenging. This is because the glycemic thresholds for hypoglycemia symptoms tend to shift: they decrease after recent antecedent hypoglycemia and increase in patients with poorly controlled diabetes or infrequent hypoglycemic episodes. As a result, the cutoff value for symptomatic hypoglycemia can vary from one patient to another. It is often better defined as the "serum glucose level that draws the attention of both patients and caregivers to the potential harm associated with hypoglycemia" [2]. There has been significant debate regarding the definition of a serum plasma cutoff for hypoglycemia, with values ranging between 3.3 and 3.9 mmol/L [3-7]. The ADA, Canadian Diabetes Association (CDA), and the European Medicines Agency (EMA) define hypoglycemia as a serum glucose level of ≤3.9 mmol/L [8], which is the cutoff value used in this study.

The administration of a high-concentration glucose solution is the standard initial treatment for symptomatic hypoglycemia. The most commonly used solution is 50 mL of 50% dextrose (D50), which contains 25 g of glucose [9]. The immediate reversal of presenting symptoms following D50 administration confirms the diagnosis of hypoglycemia [8]. Due to the unpredictability of glucose metabolism in hypoglycemic patients, it is clinically important to understand how quickly administered dextrose raises serum glucose levels, to what extent, and how fast it may drop again. This information helps estimate the risk of rebound hyperglycemia and whether a second dose of 50 mL D50 may be required to prevent further hypoglycemia and maintain normal serum glucose levels.

The objectives of this study were to observe changes in serum glucose levels following the administration of 50 mL D50 to adult hypoglycemic patients. The study also aimed to estimate the peak effect and duration of D50 as well as determine the rate of rebound hyperglycemia.

## Materials and methods

**Figure 1:**
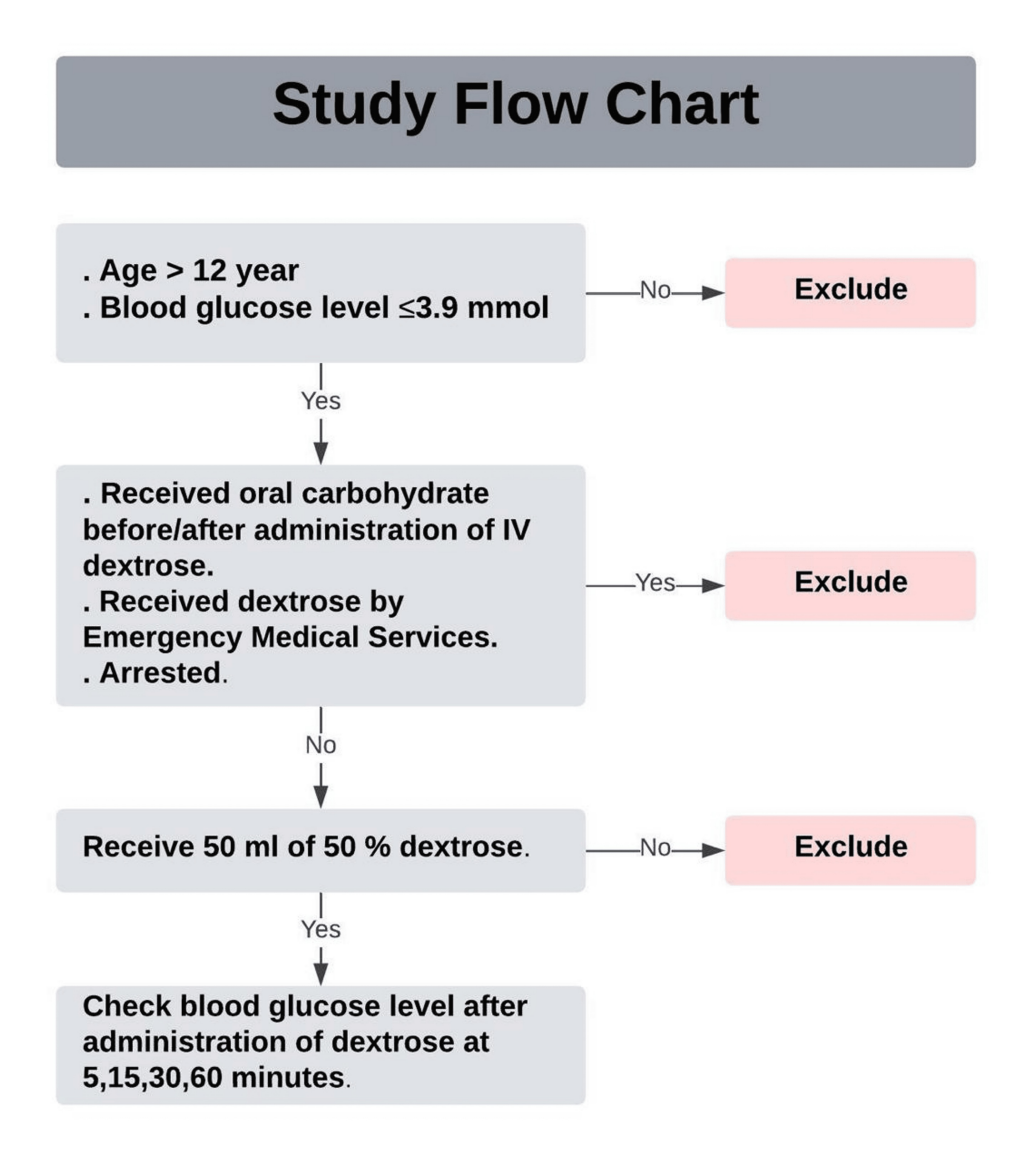
Study flowchart (patient selection)

This prospective, multicenter observational study was conducted at four emergency departments (two tertiary care hospitals and two secondary care hospitals) in Oman over a three-year period, from May 1, 2019, to April 30, 2022. The study included all patients over the age of 12 who presented to the emergency departments with documented low serum glucose levels, regardless of whether they had diabetes. Hypoglycemia was defined as a serum glucose level of ≤3.9 mmol/L based on the ADA's definition, irrespective of clinical signs, symptoms, medical history, level of consciousness, or clinical presentation. The decision to administer the D50 solution was at the discretion of the treating physician and was not part of the study protocol. Only patients who received the D50 solution for the management of their hypoglycemia were included in the analysis. Patients who were brought in with cardiac arrest, received intravenous dextrose prior to arrival at the emergency department, received oral carbohydrates before D50 administration or within the predetermined time intervals for serum glucose measurement, or did not receive 50 mL of D50 solution for any reason were excluded from the study (Figure 1).

After the administration of 50 mL D50, all patients underwent subsequent bedside serum glucose measurements using a glucometer device at predetermined time intervals to observe changes in serum glucose levels. The first reading was taken prior to D50 administration, followed by four readings post-administration at five, 15, 30, and 60 minutes. These time intervals were chosen to determine the peak effect and establish the duration of action of a single dose of 50 mL D50.

Data were collected and analyzed using the Epidata program (Buenos Aires, Argentina). The independent sample t-test, one-way ANOVA, and Pearson's chi-squared test were used for statistical analysis, and the data were presented using linear graphs.

## Results

Initially, 111 patients were included in the study. However, nine patients were excluded: one patient received juice from the Emergency Medical Service (EMS) before arrival at the emergency department, one received a high-concentration glucose solution via a nasogastric tube immediately after receiving intravenous 50 mL D50, three patients started oral carbohydrate intake within the predetermined time intervals, and three patients had incomplete data (undocumented glucose measurements on the data collection sheet). Additionally, one case was excluded due to failure to adhere to the study protocol for subsequent serum glucose measurement times.

**Figure 2:**
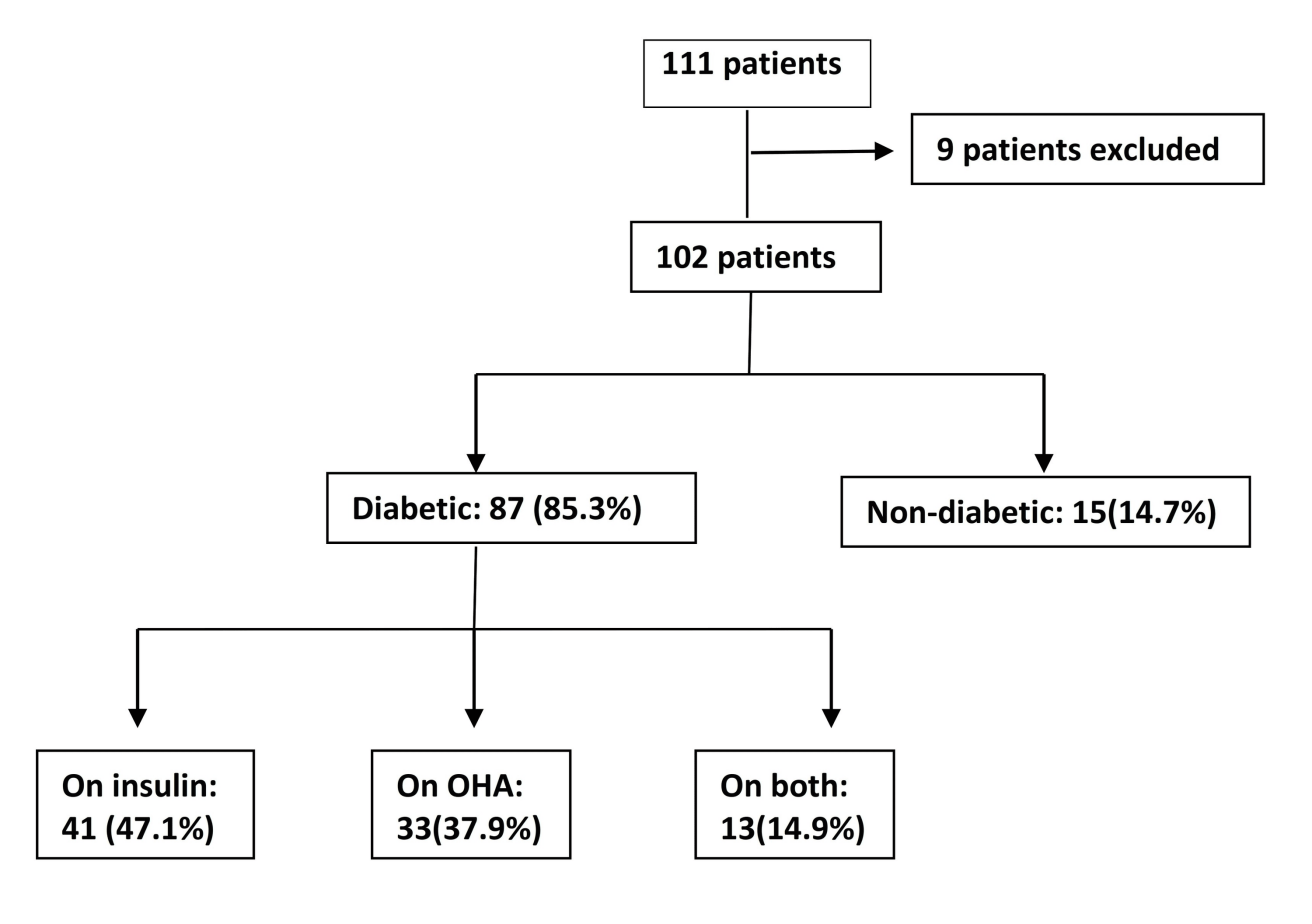
Study population enrollment algorithm OHA: oral hypoglycemic agent

A total of 102 patients were eligible for enrollment based on the study protocol. Of these, 53 individuals (52%) were female, and the mean age was 66.4 years (Figure 2). The majority of the patients were diabetic, accounting for 87 (85.3%), while non-diabetic patients made up 15 (14.7%). Among the diabetic patients, 41 (47.1%) were using insulin alone to control their blood sugar, 33 (37.9%) were using oral hypoglycemic agents (OHAs) alone, and 13 (14.9%) were on both insulin and OHAs.

**Figure 3:**
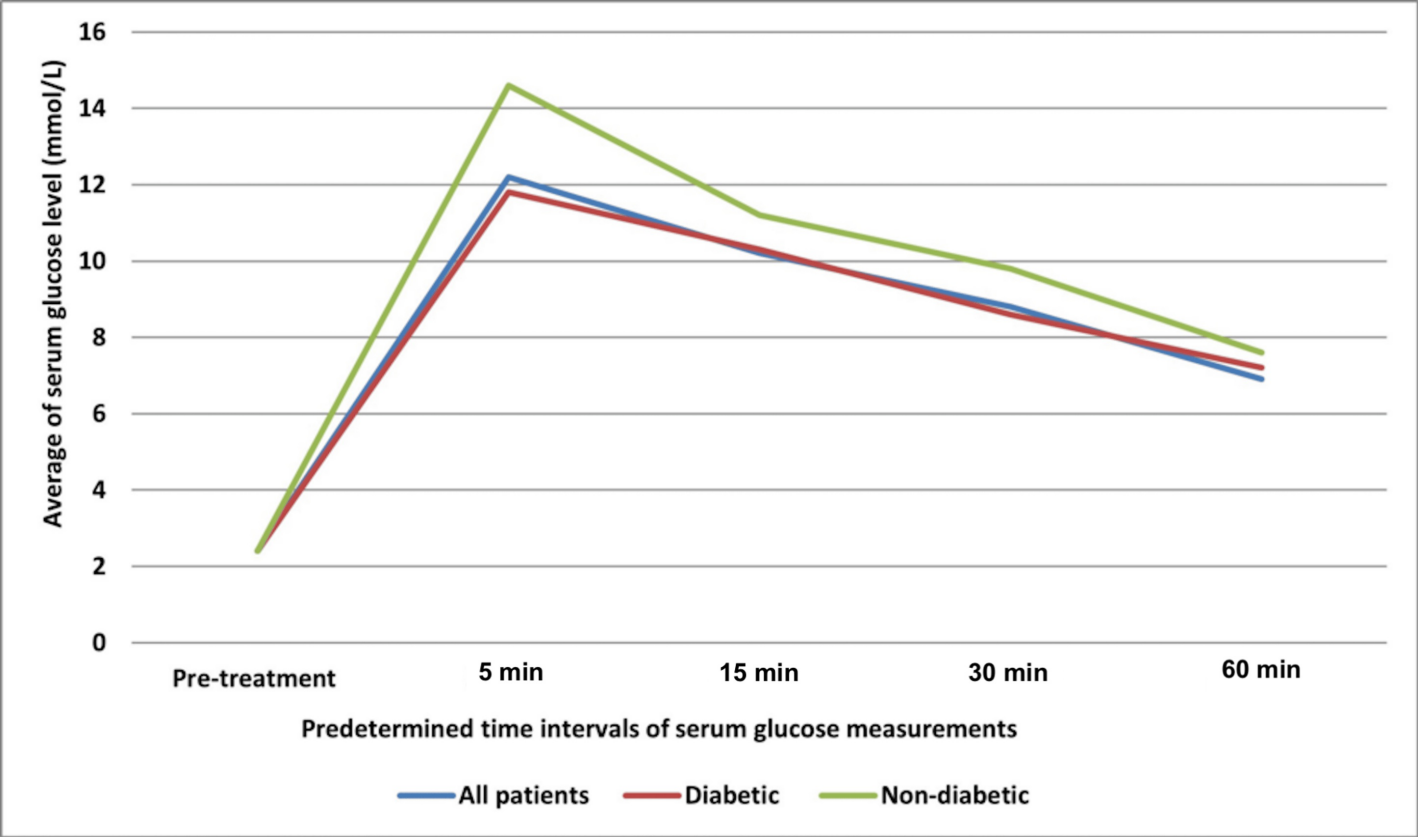
Mean serum glucose levels at predetermined time intervals

The mean pre-treatment serum glucose level for all patients (diabetic and non-diabetic) was 2.4 mmol/L, with a minimum reading of 1.0 mmol/L and a maximum reading of 3.9 mmol/L. The peak serum glucose level was observed five minutes after the administration of D50, with a mean value of 12.2 mmol/L (Figure 3). The highest serum glucose reading (31.5 mmol/L) was observed in one diabetic patient at 30 minutes from the diabetic group.

The mean serum glucose readings at each time interval were similar for both diabetic and non-diabetic groups, except at five minutes, where the non-diabetic patients had slightly higher readings than the diabetic patients (P = 0.008; see Table 1). A subgroup analysis of diabetic patients (those on OHAs, insulin, and both insulin and OHAs) showed similar mean serum glucose levels across the three subgroups (Table 2).

**Table 1:** Mean serum glucose levels at each time interval among all patients (N = 87) *Two patients were not included in this time interval analysis as they received a second dose of D50 at 30 minutes. P-values were calculated using the independent sample t-test.

Time Interval	Categories	No. of Patients	Mean	Minimum	Maximum	Std. Deviation	T-value	P-value
Pre-treatment serum glucose level	All patients	102	2.4	1.0	3.9	0.68	0.000	1.000
	Diabetic	87	2.4	1.0	3.9	0.68		
	Non-diabetic	15	2.4	1.0	3.7	0.69		
Serum glucose level at 5 minutes	All patients	102	12.2	4.5	22.6	3.8	-2.706	0.008
	Diabetic	87	11.8	4.5	19.0	3.49		
	Non-diabetic	15	14.6	6.0	22.6	4.8		
Serum glucose level at 15 minutes	All patients	102	10.4	3.1	17.5	2.8	-1.152	0.252
	Diabetic	87	10.3	3.1	17.5	2.82		
	Non-diabetic	15	11.2	5.6	16.9	2.63		
Serum glucose level at 30 minutes	All patients	102	9	3.6	31.5	3.30	-0.972	0.334
	Diabetic	87	8.9	3.6	31.5	3.34		
	Non-diabetic	15	9.8	5.1	17.3	3.14		
Serum glucose level at 60 minutes	All patients	100*	7.3	2.3	17.0	2.57	-0.553	0.582
	Diabetic	85	7.2	2.3	17.0	2.56		
	Non-diabetic	15	7.6	3.5	12.9	2.73		

**Table 2:** The mean readings of serum glucose level at each time interval among diabetic patients (sub-group analysis). *Two patients received a second dose of D50 at 30 minutes and were not included in subsequent analysis. P-values were calculated using the one-way ANOVA test. OHA: oral hypoglycemic agent

Time Interval	Categories	No. of Patients	Mean	Minimum	Maximum	Std. Deviation	F-value	P-value*
Pre-treatment serum glucose level	All patients	87	2.4	1.0	3.9	0.68	2.14	0.124
	On insulin	41	2.2	1.0	3.9	0.76		
	On OHA	33	2.5	1.2	3.8	0.63		
	On both	13	2,5	1.9	3.4	0.46		
Serum glucose level at 5 minutes	All patients	87	11.8	4.5	19.0	3.49	2.68	0.074
	On insulin	41	11.1	4.5	18.8	3.7		
	On OHA	33	12.9	7.6	18.9	3.2		
	On both	13	11.3	5.2	19.0	3.1		
Serum glucose level at 15 minutes	All patients	87	10.3	3.1	17.5	2.82	0.64	0.533
	On insulin	41	19.7	3.1	16.4	2.75		
	On OHA	33	10.7	6.0	17.5	2.84		
	On both	13	10.3	6.2	15.6	2.91		
Serum glucose level at 30 minutes	All patients	87	8.6	3.6	31.5	3.33	0.53	0.588
	On insulin	41	8.6	3.6	31.5	4.22		
	On OHA	33	8.9	2.5	10.7	2.31		
	On both	13	9.7	7.0	14.8	2.21		
Serum glucose level at 60 minutes	All patients	85*	7.2	2.3	17.0	2.56	1.08	0.346
	On insulin	39	7.3	2.3	17.0	2.90		
	On OHA	33	6.8	2.5	10.7	2.11		
	On both	13	8.0	2.3	12.7	2.43		

Rebound hyperglycemia (serum glucose level >11.1 mmol/L) was observed in both diabetic and non-diabetic groups, with a higher prevalence in the non-diabetic group (73.3%) compared to the diabetic group (56.3%). The highest prevalence of rebound hyperglycemia occurred five minutes post-D50 administration, affecting 58.8% of patients. Serum glucose levels returned to a euglycemic state (<11.1 mmol/L) in 86.3% of patients within 30 minutes and in 92.3% within 60 minutes (Table 3).

**Table 3:** Prevalence of rebound hyperglycemia among each group P-values were calculated using Pearson's chi-squared test.

Time Interval	All Patients (N = 102)	Diabetic (N = 87)	Non-diabetic (N = 15)	χ2 Statistics	P-value*
At 5 minutes	60 (58.8%)	49 (56.3%)	11 (73.3%)	0.91	0.341
At 15 minutes	36 (35.3%)	27 (31.0%)	9 (60.0%)	3.52	0.061
At 30 minutes	14 (13.7%)	11 (12.6%)	3 (20.0%)	0.50	0.478
At 60 minutes	8 (7.8%)	6 (6.9%)	2 (13.3%)	0.36	0.549

Among all patients, eight (7.8%) had serum glucose levels less than 3.9 mmol/L during the one-hour period following D50 administration. Of these, two did not receive a second dose of D50, as this decision was made by the treating physicians and was not part of the study protocol. Six (5.9%) patients received a second dose of 50 mL D50, all of whom were diabetic. Three of these patients were on OHAs, and the other three were using insulin alone. Four of these patients received the second dose 60 minutes after the first dose, while two patients received the second dose 30 minutes after the first. One of these patients had a serum glucose level of 3.1 mmol/L at 30 minutes and did not receive a second dose at that time. Instead, the second dose was administered at 60 minutes when their serum glucose level had dropped to 2.3 mmol/L, as per the treating physician's decision. All eight patients requiring a second dose were from the diabetic group, while no patients in the non-diabetic group required a second dose of D50.

## Discussion

This study aimed to observe changes in serum glucose levels following the administration of 50 mL D50 to hypoglycemic patients, assess the prevalence of rebound hyperglycemia episodes, and estimate the peak and duration of effect of a single dose of D50 in adults after a hypoglycemic episode.

As expected, diabetic patients (85.3%) were more prone to hypoglycemia compared to non-diabetic patients (14.7%), likely due to the side effects of diabetic medications and the tight blood sugar control typically required in this population. A large retrospective study has demonstrated that diabetic patients are significantly more prone to hypoglycemia than their non-diabetic counterparts due to altered counter-regulatory mechanisms and the effects of pharmacologic interventions [8]. Similarly, a study by Kagansky et al. (2003) identified diabetes as a primary risk factor for hypoglycemia in hospitalized patients, with an increased prevalence among those with comorbid conditions such as chronic kidney disease [1]. These findings align with the current study, emphasizing the heightened vulnerability of diabetic patients to hypoglycemia. Chronic complications of diabetes mellitus, such as chronic kidney disease and infections (particularly sepsis), further increase the risk of hypoglycemia [10-12], although these complications were not addressed in this study.

The observed peak effect of 50 mL D50 within five minutes of administration aligns with the established pharmacodynamics of intravenous glucose (Figure 1). The rapid elevation in serum glucose levels reflects the immediate bioavailability of glucose when administered intravenously, bypassing first-pass metabolism. This rapid response has been corroborated by studies of glucose metabolism, which highlight the near-instantaneous increase in plasma glucose levels following intravenous dextrose administration, particularly in hypoglycemic states [13]. Additionally, the subsequent decline in serum glucose levels observed in this study is consistent with the physiological uptake and utilization of glucose by insulin-sensitive tissues, as described in metabolic studies [14].

The sustained serum glucose levels (>3.9 mmol/L) observed in non-diabetic patients after a single dose of D50, without the need for further doses, reflect the efficient regulatory mechanisms of glucose homeostasis in this population. Studies on glucose dynamics in healthy individuals similarly highlight the ability of non-diabetic individuals to maintain prolonged euglycemia following acute hypoglycemic episodes, even after the administration of high-concentration glucose [13]. This contrasts with diabetic patients, whose impaired glucose regulation contributes to a shorter duration of glycemic control [14]. A prior study from 1998 on euglycemic healthy volunteers reported a more rapid return to baseline glucose levels at 30 minutes [5]. This difference might be attributed to the different metabolic states of the study populations.

The duration of the effect of a single dose of D50 was shorter among diabetic patients, likely due to the continued influence of glucose-lowering medications or their underlying illnesses. This finding is consistent with recent studies, which suggest that the pharmacokinetics of glucose metabolism in diabetic patients can be altered due to insulin resistance or ongoing treatment with antidiabetic medications [15]. In the diabetic patient group, eight patients had serum glucose levels ≤3.9 mmol/L, and six of them required a second dose of D50. Although the number of patients is small, this observation aligns with findings from other studies, which reported that diabetic patients are more likely to experience recurrent hypoglycemia due to their altered glucose homeostasis [16].

Given the potential complications that may arise from hypoglycemia, including seizures or loss of consciousness, it is crucial for the treating physician to remain vigilant in monitoring glucose levels and responding promptly to any signs of hypoglycemia. Repeated doses of 50 mL D50 may be necessary for diabetic patients who present with hypoglycemia, particularly if the patient has not started oral feeding or has been initiated on an infusion of glucose-containing intravenous fluid. Recent studies have shown that diabetic patients who do not receive adequate glucose supplementation after initial treatment remain at risk of further hypoglycemic episodes, requiring additional glucose administration [17]. Moreover, failure to initiate oral feeding or glucose infusion promptly can exacerbate the recurrence of hypoglycemia [18]. These recommendations are also supported by recent guidelines, which emphasize the importance of early intervention and the administration of additional glucose to prevent further complications [19].

A previous review highlighted the importance of individualized treatment plans for diabetic patients with hypoglycemia, as responses to glucose administration can vary based on the patient's overall health status and concurrent medications [20].

Rebound hyperglycemia was observed in over half of the patients in this study, with 58.8% experiencing hyperglycemia within five minutes of D50 administration and 13.7% remaining hyperglycemic 30 minutes post-administration. This finding aligns with previous research indicating that rapid administration of dextrose solutions can lead to transient hyperglycemia, particularly in patients with impaired glucose regulation [21-23]. In contrast, a previous study reported a significantly lower incidence of overcorrection, with only 6.8% of patients affected [24]. However, this study defined hyperglycemia using a lower cutoff value of 8.3 mmol/L compared to the 11.1 mmol/L applied in the current study. This discrepancy in criteria suggests that the observed rate of rebound hyperglycemia in the present study may have been even higher had the same threshold been used. Rebound hyperglycemia is more likely to occur in patients with impaired insulin secretion, a factor not always controlled for in prior research [25,26]. Therefore, individualized treatment plans are essential to effectively manage glucose levels in diabetic patients.

Study limitations

One of the limitations of this study was the response of treating physicians in the emergency departments, which fell below expectations, leading to the exclusion of many patients with low serum glucose levels. Including these patients in the study could have potentially altered the results. The study period was extended to three years due to the concurrent COVID-19 outbreak and the aforementioned limitations. Additionally, the study did not account for the clinical presentation of patients (symptomatic, asymptomatic, or unconscious) or specify the types of OHAs used among diabetic patients. Information about comorbidities, such as chronic kidney disease or malignancy, was not considered in the data analysis, which may have impacted the generalizability of the findings. Finally, a 60-minute observation period may be insufficient to detect late-onset rebound hypoglycemia. We acknowledge that extended monitoring (e.g., two to four hours) would provide critical insights into its progression and clinical management. Future studies may benefit from incorporating longer observation periods to enhance understanding and optimize patient care.

## Conclusions

This study suggests that a single dose of 50 mL D50 is sufficient to restore and maintain the desired serum glucose levels in non-diabetic hypoglycemic adult patients. However, diabetic patients may require repeated doses or maintenance with dextrose-containing fluids, particularly if they have not started oral feeding. Additionally, the study highlights the risk of rebound hyperglycemia, which may warrant the use of lower-concentration dextrose fluids, such as D25 or D10, to mitigate this effect. These findings underscore the need for further randomized controlled trials to compare the efficacy of D50 with lower-concentration glucose solutions, which could provide more tailored approaches to managing hypoglycemia in both diabetic and non-diabetic patients.
